# Protective effect and mechanism of Qingfei Paidu decoction on myocardial damage mediated by influenza viruses

**DOI:** 10.3389/fphar.2024.1309682

**Published:** 2024-02-27

**Authors:** Lijuan Du, Jing Zhao, Nanxi Xie, Huangze Xie, Jiating Xu, Xiaoming Bao, Yingsong Zhou, Hui Liu, Xiao Wu, Xin Hu, Tianyi He, Shujun Xu, Yuejuan Zheng

**Affiliations:** ^1^ Department of Physiology and Pathophysiology, Health Science Center, Ningbo University, Ningbo, China; ^2^ Faculty of Physical Education, Ningbo University, Ningbo, Zhejiang, China; ^3^ Institute of Interdisciplinary Integrative Medicine Research, Shanghai University of Traditional Chinese Medicine, Shanghai, China; ^4^ The Research Center for Traditional Chinese Medicine, Shanghai Institute of Infectious Diseases and Biosecurity, Shanghai University of Traditional Chinese Medicine, Shanghai, China; ^5^ Department of Cardiology, Ningbo No.2 Hospital, Ningbo, Zhejiang, China; ^6^ Center for Traditional Chinese Medicine and Immunology Research, School of Integrative Medicine, Shanghai University of Traditional Chinese Medicine, Shanghai, China

**Keywords:** Qingfei Paidu decoction, myocardial damage, influenza virus, necroptosis, HIF-1α, coronavirus disease 2019

## Abstract

**Introduction:** Significant attention has been paid to myocardial damage mediated by the single-stranded RNA virus. Qingfei Paidu decoction (QFPDD) has been proved to protect the damage caused by the influenza virus A/PR/8/1934 (PR8), but its specific mechanism is unclear.

**Methods:** Molecular biological methods, together with network pharmacology, were used to analyze the effects and underlying mechanism of QFPDD treatment on PR8-induced myocardial damage to obtain insights into the treatment of COVID-19-mediated myocardial damage.

**Results:** Increased apoptosis and subcellular damage were observed in myocardial cells of mice infected by PR8. QFPDD treatment significantly inhibited the apoptosis and subcellular damage induced by the PR8 virus. The inflammatory factors IFN-β, TNF-α, and IL-18 were statistically increased in the myocardia of the mice infected by PR8, and the increase in inflammatory factors was prevented by QFPDD treatment. Furthermore, the expression levels or phosphorylation of necroptosis-related proteins RIPK1, RIPK3, and MLKL were abnormally elevated in the group of infected mice, while QFPDD restored the levels or phosphorylation of these proteins. Our study demonstrated that HIF-1α is a key target of QFPDD in the treatment of influenza virus-mediated injury. The HIF-α level was significantly increased by PR8 infection. Both the knockdown of HIF-1α and treatment of the myocardial cell with QFPDD significantly reversed the increased inflammatory factors during infection. Overexpression of HIF-1α reversed the inhibition effects of QFPDD on cytokine expression. Meanwhile, seven compounds from QFPDD may target HIF-1α.

**Conclusion:** QFPDD can ameliorate influenza virus-mediated myocardial damage by reducing the degree of cell necroptosis and apoptosis, inhibiting inflammatory response and the expression of HIF-1α. Thus, our results provide new insights into the treatment of respiratory virus-mediated myocardial damage.

## 1 Introduction

Single-stranded RNA viruses, for example, influenza virus and coronavirus disease 2019 (COVID-19), have emerged as a global pandemic. The influenza virus is responsible for nearly 500,000 deaths worldwide every year (Corrales-Medina et al., 2012). While the molecular and epidemiological effects of single-stranded RNA viral infection in lungs are well documented, its molecular effects in other organs need further demonstration (Filgueiras-Rama et al., 2021). However, there is still lack of effective treatment.

Increasing attention has been paid to myocardial damage mediated by single-stranded RNA viruses. Studies have shown that patients with viral infection are more likely to develop cardiac complications, which are usually associated with increased short-term mortality (Corrales-Medina et al., 2012). Fatalities from typical seasonal influenza have shown myocarditis in up to 48% of all autopsies (Ukimura et al., 2012). Virus-induced myocardial damage might be caused by direct damage of cardiomyocytes, inflammatory response, and hypoxia-induced cardiomyocyte injury (Takahashi et al., 2018). Effective treatment may be directed at the synergistic effects of multiple targets.

Qingfei Paidu decoction (QFPDD), a traditional Chinese medicine formula commonly used for treating damage caused single-stranded RNA viruses at various stages, has been demonstrated to be safe and effective (An et al., 2021; Bhat et al., 2021; Yang et al., 2021). However, the specific mechanism of its protective effects needs further investigation. Earlier studies have shown that either QFPDD or its components have protective effects on hypoxia-mediated myocardial cell damage (Ren et al., 2021). Additionally, our previous study had revealed that the compounds of QFPDD were present in the cardiac tissue of mice after the administration of QFPDD (Liu et al., 2021), suggesting that QFPDD might exert its protective effects through inhibiting cardiac damage induced by viral infection. However, the mechanisms that underlie the protective effects of QFPDD on influenza virus-induced myocardial damage are unclear. Therefore, our study aimed to explore the protective effects and mechanisms of QFPDD on influenza virus-mediated myocardial damage to obtain insights into the treatment of single-stranded RNA virus-mediated myocardial damage.

## 2 Materials and methods

### 2.1 Reagents

The lyophilized powder of QFPDD used in the experiment was provided by Prof. Guang-Bo Ge from the Institute of Interdisciplinary Integrative Medicine Research, Shanghai University of Traditional Chinese Medicine, which contained *Radix Bupleuri (Chaihu), Pinelliae Rhizoma (Banxia), Scutellaria baicalensis Georgi (Huangqin), Glycyrrhizae radix et rhizome (Gancao), Zingiber officinale (Shengjiang), Polyporus (Zhuling), Alismatis Rhizoma (Zexie), Poria (Fuling), Cinnamomi Ramulus (Guizhi), Ephedrae herba (Mahuang), Atractylodes Macrocephala Rhizoma (Baizhu), Armeniacae semen amarum (Xingren), Belamcanda chinensis (Shegan), Pinellia ternata (Banxia), Asarum sieboldii (Xixin), Aster tataricus (Ziwan), Tussilago farfara (Donghua), Schisandra chinensis (Wuweizi), Ziziphus jujube (Dazao)*, and a mineral drug *Gypsum Fibrosum (Shigao)*. It was stored in a dry tower in an airtight manner after production. QFPDD was ground into lyophilized powder according to the proportion of clinical dosage. The dosage of each mouse was calculated according to the clinical dosage, 6 g/kg. QFPDD was diluted to a concentration of 0.6 g/mL with ultra-pure water, and 200 μL was given by gavage to each rat weighing approximately 20 g (Ye et al., 2023). Oseltamivir (OSV) phosphate granules (YiChang HEC ChangJiang Pharmaceutical Co., Ltd., Hubei, China) were diluted to 3 mg/mL with ultra-pure water (Wu and McGoogan, 2020).

### 2.2 Animals and infection

All animals were housed in a specific pathogen-free facility at the Animal Care Facility of the Institution Pasteur of Shanghai, Chinese Academy of Sciences, and were approved by the Institutional Animal Care and Use Committee. SPF-grade C57BL/6J female mice (16–18 g) were purchased from the Joint Ventures Sipper BK Experimental Animal Company (Shanghai, China) and reared in a quarantine room for at least 1 week. All animal experiments were conducted in an ABSL-2 laboratory at the Chinese Academy of Sciences, and the ethical registration number was A2021-L027. The mice were randomly divided into four groups: the control group (control), PR8-infected group (model), oseltamivir-treated group (PR8+OSV), and QFPDD-treated group (PR8+QFPDD). The infective mouse model of influenza was created by intranasal administration of 50 μL PR8 provided by Professor Robert G. Webster (Jude Children’s Research Hospital, Memphis, United States) at a dose of 4 × 10^5^ PFU/mL. The mice were given oseltamivir (30 mg/kg) or QFPDD (6 g/kg) in 0.2-mL doses once a day by gavage. The first dose of drugs was administered within 2 h on the day of infection. The control and model group mice received a similar volume of the vehicle instead of the drug. On day 3 after PR8 infection, all mice were euthanized 2 h after the last gavage. The hearts were separated and used for pathological observation.

### 2.3 Histology

Hematoxylin and eosin (HE) staining was carried out to assess the pathological damage of the myocardium. Myocardial tissues fixed in 4% formaldehyde were embedded in paraffin and sliced at a thickness of approximately 4–5 μm for HE staining. The morphological change in slices was observed using a microscope and imaging analysis system.

### 2.4 Transmission electron microscopy

The hearts were cut into blocks of 0.5 × 1 × 1 mm. After fixing in 4% glutaraldehyde containing 0.1 M cacodylate buffer for 1 h at 4°C, they were fixed in 1% osmium tetroxide in the above buffer for 1 h at room temperature. The samples were embedded in Epon after being dehydrated through a graded series of ethanol. Ultrathin sections were stained with 2% uranyl acetate and lead citrate. Transmission electron microscopy (TEM) was performed using a Hitachi HT-7800 transmission electron microscope (HITACHI, HT-7800, Japan).

### 2.5 TUNEL staining

The myocardial tissues were fixed in 4% paraformaldehyde for at least 24 h for paraffin embedding. They were permeabilized with 0.5% Triton X-100 and blocked by 2% BSA in PBS for 30 min. The myocardial sections were incubated with the TUNEL reaction buffer (Uelandy, China) for 40 min. DAPI was added to counterstain the nuclei. Then, the sections were captured using a fluorescent microscope**.**

### 2.6 Quantitative RT-PCR

The total RNA was extracted from the myocardial tissues or cells and reverse-transcribed into cDNA following standard experimental procedures. The relative transcript level of INF-β, TNF-α, IL-18, IL-6, and IFN-γ was detected by quantitative PCR using LightCycle 480VR II PCR (Roche, Switzerland) with specific primers (Table 1).

**TABLE 1:** Nucleotide sequences, amplification sites, GenBank accession numbers, and amplimer sizes for each primer pair.

Gene	Primer	Nucleotide sequence (5′→3′)	GenBank ID	Amplimer sizes
Acta2	Forward	GTC​CCA​GAC​ATC​AGG​GAG​TAA	NM_007392	102
	Reverse	TCG​GAT​ACT​TCA​GCG​TCA​GGA		
IL-6	Forward	TAG​TCC​TTC​CTA​CCC​CAA​TTT​CC	NM_031168	76
	Reverse	TTG​GTC​CTT​AGC​CAC​TCC​TTC		
IL-18	Forward	GAC​TCT​TGC​GTC​AAC​TTC​AAG​G	NM_008360	169
	Reverse	CAG​GCT​GTC​TTT​TGT​CAA​CGA		
IFN-β	Forward	TGG​GTG​GAA​TGA​GAC​TAT​TGT​TG	NM_010510	109
	Reverse	CTC​CCA​CGT​CAA​TCT​TTC​CTC		
IFN-γ	Forward	ACA​GCA​AGG​CGA​AAA​AGG​ATG	NM_008337	106
	Reverse	TGG​TGG​ACC​ACT​CGG​ATG​A		
TNF-α	Forward	AAG​CCT​GTA​GCC​CAC​GTC​GTA	NM_013693	122
	Reverse	GGC​ACC​ACT​AGT​TGG​TTG​TCT​TTG		
HIF-1α	Forward	GAT​GAC​GGC​GAC​ATG​GTT​TAC	NM_010431	155
	Reverse	CTC​ACT​GGG​CCA​TTT​CTG​TGT		
GAPDH	Forward	GAG​AAA​CCT​GCC​AAG​TAT​GAT​GAC	NM_002046.7	85
	Reverse	AGA​GTG​GGA​GTT​GCT​GTT​GAA​G		

### 2.7 Western blot assay

The tissues were homogenized in RIPA lysis buffer (Beyotime, China) containing a protease inhibitor cocktail (one tablet for every 50 mL of RIPA lysis buffer, Roche) and a phosphorylase inhibitor cocktail (one tablet for every 50 mL of RIPA lysis buffer, Roche) and then crushed by ultrasound for 10 min using an ultrasonic cell disruptor (Banoelin, Germany). After being adequately crushed, the lysates were centrifuged at 13,200 rpm for 30 min at 4 °C, and the supernatant fraction was used for Western blot assay. The protein concentration in the supernatant fraction was determined using the BCA Protein Assay Kit (Beyotime, China). Equal amounts of soluble protein (25 μg) were separated by 8% SDS-PAGE (Beyotime, China) and transferred onto polyvinylidene fluoride (PVDF) membranes (0.45 μm; Millipore, United States). After blocking with 5% BSA blocking buffer for 1 h, the membranes were incubated with rabbit anti-MLKL (1:1,000; Cell Signaling, Cat# 37705S, China), anti-phospho-MLKL (1:1,000; Cell Signaling, Cat# 18640, China), the anti-alpha subunit of hypoxia-inducible factor-1 (HIF-1α) (1:1,000; Cell Signaling, Cat# 36169, China), anti-RIPK1 (1:1,000; Cell Signaling, Cat# 3493S, China), anti-phospho-RIPK1 (1:1,000; Cell Signaling, Cat# 38662S, China), anti-RIPK3 (1:1,000; Cell Signaling, Cat# 95702S, China), anti-phospho-RIPK3 (1:1,000; Cell Signaling, Cat# 91702S, China), and anti-beta actin antibody (1:10,000; Abways, Cat# AB 2001) at 4 °C overnight. The membranes were then incubated with an HRP-linked anti-rabbit antibody (1:5,000; Affinity, Cat# #S0001, China), respectively. Target bands were detected and quantified with BeyoECL Plus (1:1; Beyotime) using Amersham Imager 600 (GE Healthcare Life Sciences, United States).

### 2.8 Putative targets for QFPDD compounds in the myocardium

Earlier experiments conducted by our team identified 77 compounds from the myocardium of mice after the administration of QFPDD (Wu and McGoogan, 2020), with a peak area in the region of [0.6 × 10^6^, 667.87 × 10^6^]. We took the compounds having the highest content in the heart (peak area >40 × 10^6^) as the main active ingredients present in the myocardium for further study. This resulted in the identification of 21 compounds (Supplementary Table S1). For each of the 21 compounds, we collected or predicted its putative targets from six databases and web servers: the Herbal Ingredients’ Targets (HIT) platform (Yan et al., 2020), Encyclopedia of Traditional Chinese Medicine (ETCM) (Xu et al., 2019), High-throughput Experiment- and Reference-guided Database (HERB) of traditional Chinese medicine (Fang et al., 2021), DrugBank (Wishart et al., 2018), D3Dockin (Shi et al., 2020), and TargetNet (Yao et al., 2016). The threshold of the confidence score was set as 0.9 to filter the compound–protein interactions predicted by TargetNet. A total of 771 distinct targets for the 21 QFPDD compounds were collected from six databases and web servers.

Then, we used the binomial cumulative distribution to determine if an identified target was more closely related to the pharmacological effects of the formula than would be expected by chance (Gu et al., 2022). In addition, if a compound–gene pair was identified from at least two of the six sources, the gene was also considered a confident target of the corresponding compound. Using this approach, we filtered 426 targets with high confidence. Last, we limited our study to target genes expressed in cardiomyocytes. These genes were screened by datasets “Normal tissue data” and “RNA single-cell-type tissue cluster data” of the R package “HPAanalyze” (Tran et al., 2019). The screening criteria were as follows: (1) tissue = heart muscle and cell type = cardiomyocytes; (2) for a gene in the dataset “RNA single-cell-type tissue cluster data,” its expression level satisfies protein transcripts per million (ptpm)>=1. Finally, 289 of the 426 targets were expressed in cardiomyocytes (Supplementary Table S2). These 289 proteins were considered QFPDD targets in cardiomyocytes for further analysis.

### 2.9 Genes associated with hypoxic myocardial injury and COVID-19

Genes associated with hypoxic myocardial injury (HMI) were collected from the GeneCards database by the keyword “hypoxic myocardial injury” and relevance score >2.

Genes associated with COVID-19 were collected from two sources: the GeneCards database by the keyword “COVID-19” and Supplementary Material of the work of Li et al. (2023), where human proteins that were physically associated with SARS-CoV-2 proteins were identified using affinity purification mass spectrometry. Genes in the intersection set of the two sources, or relevance score >1 in the GeneCards database, were selected as COVID-19-associated genes.

In this way, we collected 1,092 and 1,128 genes which were associated with COVID-19 and HMI, respectively (Supplementary Tables S3, S4). To assess the relevance of each gene to COVID-19-related hypoxic myocardial injury, we calculated a disease relevance score for each gene. This score was determined by summing up the relevance scores for both COVID-19 and HMI.

### 2.10 Network construction and analysis for QFPDD targets

The R package “clusterProfiler” version 4.0 was used to perform functional annotation for target genes (Wu et al., 2021). The genomic transcriptional regulatory network of human beings was downloaded from the TRRUST database version 2 (Han et al., 2018). It was used as a background network for the construction of a transcriptional regulatory network of QFPDD targets to genes associated with HMI and COVID-19. We used Cytoscape version 3.6.0 to construct, analyze, and visualize networks. The key targets of QFPDD for treating COVID-19-related HMI were identified by integrating topological and functional significance.

### 2.11 Cardiomyocyte treatments

Mouse cardiomyocyte (HL-1) cells were seeded into plates. For HIF-1α interference experiments, the cells were transfected with small interfering RNA (siRNA) oligos (GenePharma, China). Negative control siRNA: [sense (5′-3′): CGC​TCT​CTG​CTC​CTC​CTG​TTC, antisense (5′-3′): ATCCGTTGA CTCCGACCTTCAC] or HIF-1α-siRNA: [sense (5′-3′): GCU​GAC​CAG​UUA​CGA​UUG​UTT, antisense (5′-3′): ACA​AUC​GUA​ACU​GGU​CAG​CTT] was transfected using the siRNA Mate Transfection Reagent (GenePharma, China). For HIF-1α overexpression experiments, cells were transfected with an HIF-1α plasmid (ZsGreen-Puro, China) or control plasmid using the LipoFiter 3.0 transfection reagent (HanBio, China). During the infection of single-stranded RNA viruses, there is an amount of single-stranded RNA that could be recognized by Toll-like receptor 7 (TLR7) (To et al., 2014). TLR7 is expressed in cardiomyocytes and immune cells, which mediate the inflammatory response induced by extracellular RNA (To et al., 2014; Feng et al., 2015). Imiquimod (R837, Cat: tlrl-imqs, InvivoGen, United States), also called an imidazoquinoline compound, is a well-known agonist for TLR7. It is usually used to mimic the effect of single-stranded RNA-mediated inflammatory response (To et al., 2014; Ye et al., 2023). A measure of 7.5 μg/mL imiquimod (R837) or 10% (v/v) QFPDD serum was added to HL-1 cells 18 h after transfection. Following 18 h of incubation, the total RNA was extracted from the myocardial cell and reverse-transcribed into cDNA following standard experimental procedures. The relative transcript levels of INF-β, TNF-α, and IL-18 were detected by quantitative PCR using LightCycle 480VR II PCR (Roche, Switzerland), as previously described.

### 2.12 Molecular docking

We accessed the RCSB Protein Data Bank (PDB) database to obtain crystal structures of protein receptors (Burley et al., 2021), while three-dimensional structures of compounds were acquired from the PubChem database (Kim et al., 2021). Specifically, we obtained the crystal structure of the HIF-1α/HIF-β heterodimer (PDB ID: 4h6j; resolution: 1.52 Å) from the PDB and subsequently isolated HIF-1α (chain A) from the dimer using PyMOL (Alexander et al., 2011). The isolated PDB file of chain A was then input into Protein Plus, where we utilized the “DoGSiteScorer binding site detection” method to predict the binding site (Schöning-Stierand et al., 2022). The primary binding site prediction yielded a top-ranked pocket with center coordinates of (19.65, −9.94, −28.9) and a diameter of 20 Å, which was subsequently used to define the docking box for our analyses. Finally, we conducted the docking simulations using AutoDock Vina software in accordance with the prescribed protocol (Trott and Olson, 2010). Microarray experiments for QFPDD compounds were conducted on MCF7 cells to investigate their gene expression profiles and potential therapeutic effects.

The gene expression profiles for 16 of the compounds identified in the myocardium of mice after the administration of QFPDD (pseudoephedrine, hesperidin, baicalin, wogonoside, irisflorentin, nobiletin, wogonin, alisol O, naringin, quinic acid, neohesperidin, stachydrine, salicylic acid, tilianin, ephedrine, and amygdalin) were produced for MCF7 cells. One of the nine cell lines was used in the connectivity map (CMAP) L1000 database (Subramanian et al., 2017). The MCF7 cells were purchased from the American Type Culture Collection (ATCC). These cells were maintained in MEM/EBSS supplemented with 10% fetal bovine serum, 1 mmol/L sodium pyruvate, 0.1 mM MEM non-essential amino acids, 100 unit/mL penicillin, and 100 mg/mL streptomycin in a humidified environment under 5% CO_2_ and 95% air at 37 °C. The cells were then treated with each compound at 100 μg/mL for 12 h, and solvent (DMSO)-treated cells were used as controls. Total RNA samples were extracted from the MCF7 cells using the TRIzol reagent (Life Technologies, Carlsbad, CA, United States), and the RNA samples were purified from cells using the QIAGEN RNeasy Kit (GmBH, Germany). The cRNA products, generated from the fragmentation of double-stranded cDNA and biotin-labeled cRNA, were pooled to perform microarray experiments using the Affymetrix chip (Human U133 A 2.0) by Shanghai Biotechnology Corporation in accordance with the Affymetrix technical manual. Genes with |log2 fold change| >1 and *p* < 0.05 were considered as differentially expressed. The expression data are uploaded to Synapse and are available at: https://www.synapse.org/#!Synapse: syn25843090/files/.

### 2.13 Statistical analyses

Statistical analyses were performed using GraphPad Prism software (GraphPad Software Inc., La Jolla, CA, United States, RRID:SCR_00298). The data were presented as the mean ± SEM. Multiple group data were analyzed using one-way analysis of variance (ANOVA), followed by Tukey’s *post hoc* test. *p* < 0.05 was considered statistically significant.

## 3 Results

### 3.1 QFPDD inhibited myocardial damage mediated by PR8

**FIGURE 1:**
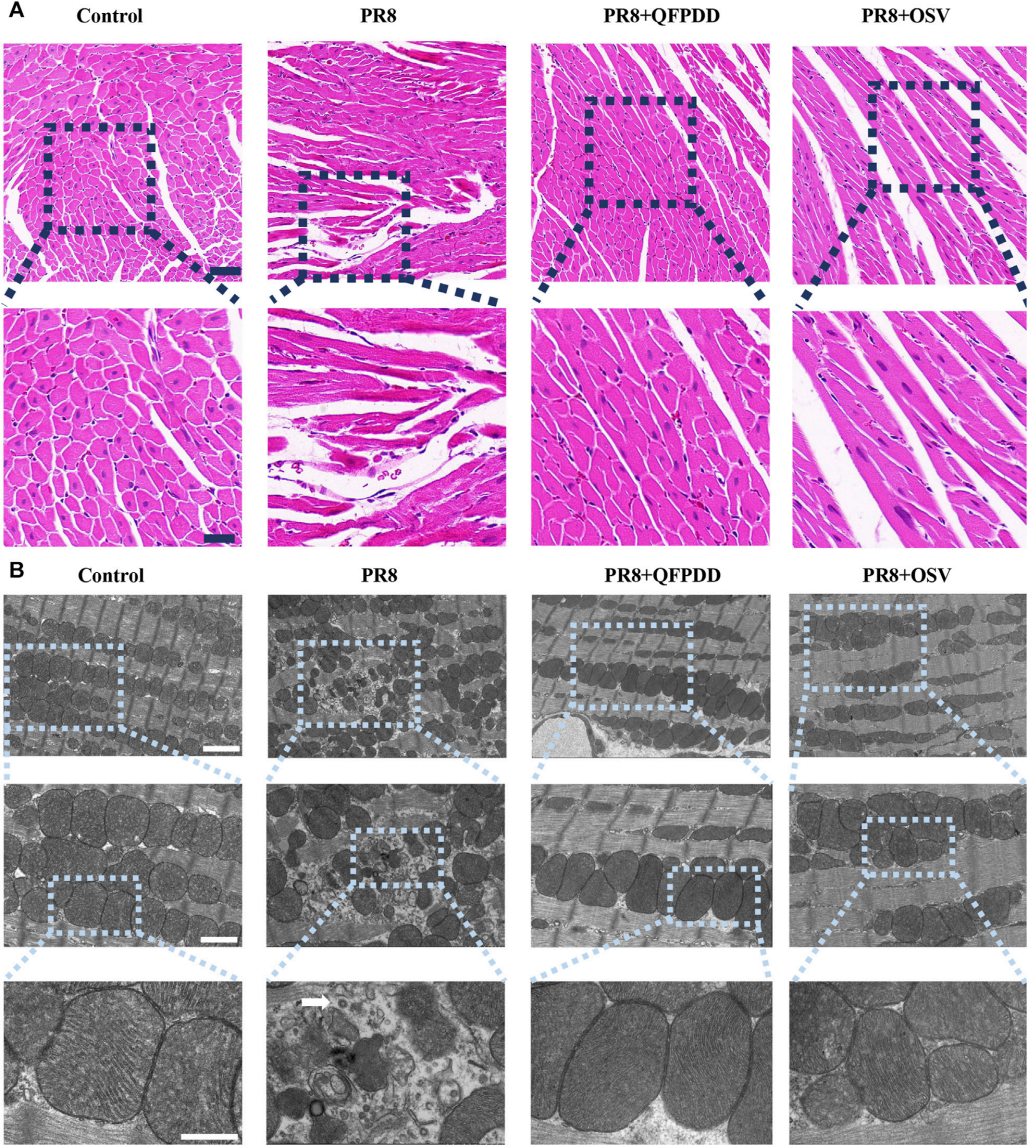
QFPDD inhibited myocardial damage mediated by PR8. **(A)** HE staining of myocardial tissue in each group. Effects of QFPDD on histopathological changes in cardiac tissues of PR8-infected mice and representative hematoxylin–eosin pathological photomicrographs of left ventricular tissues are shown. *n* = 6. Scale bar: 50 μm and 20 μm. **(B)** TEM of myocardial tissue in each group. The white arrowhead indicates a viral particle. *n* = 3. Scale bar: 2 μm, 1 µm, and 500 nm.

To test the effect of QFPDD on PR8-infected myocardial injury, we first examined the histological changes in the myocardia of mice. Oseltamivir (OSV), a drug approved for the treatment of influenza, targets the neuraminidase distributed on the surface of the influenza virus to inhibit its spread in the host and was used as an effective control (Duwe, 2017). HE staining of heart tissue showed a normal branched appearance of the myofibrillar structure and striation and continuity with adjacent myofibrils in the control group. However, myocardial fibers in PR8-infected mice were disordered and irregularly arranged with lysis and rupture compared with control groups. Basically, samples from the mice treated with QFPDD or OSV showed normal and preserved cardiomyocyte histology. In addition, histological sections from QFPDD- or OSV-treated mice showed a nearly normal myofiber structure with significant striations (Figure 1A).

To verify the protective effect of QFPDD, TEM was also performed to observe the subcellular structures of the QFPDD-treated or PR8-infected cardiomyocytes. It showed normal cellular and subcellular morphology in the control group. On the contrary, mitochondria in PR8-infected myocardial cells exhibited necrosis-like phenotypic changes, including swelling and irregular arrangement, with a few viral particles observed in the heart. QFPDD or OSV treatment reversed the subcellular damage induced by PR8. Most of the mitochondria appeared normal under TEM (Figure 1B).

**FIGURE 2:**
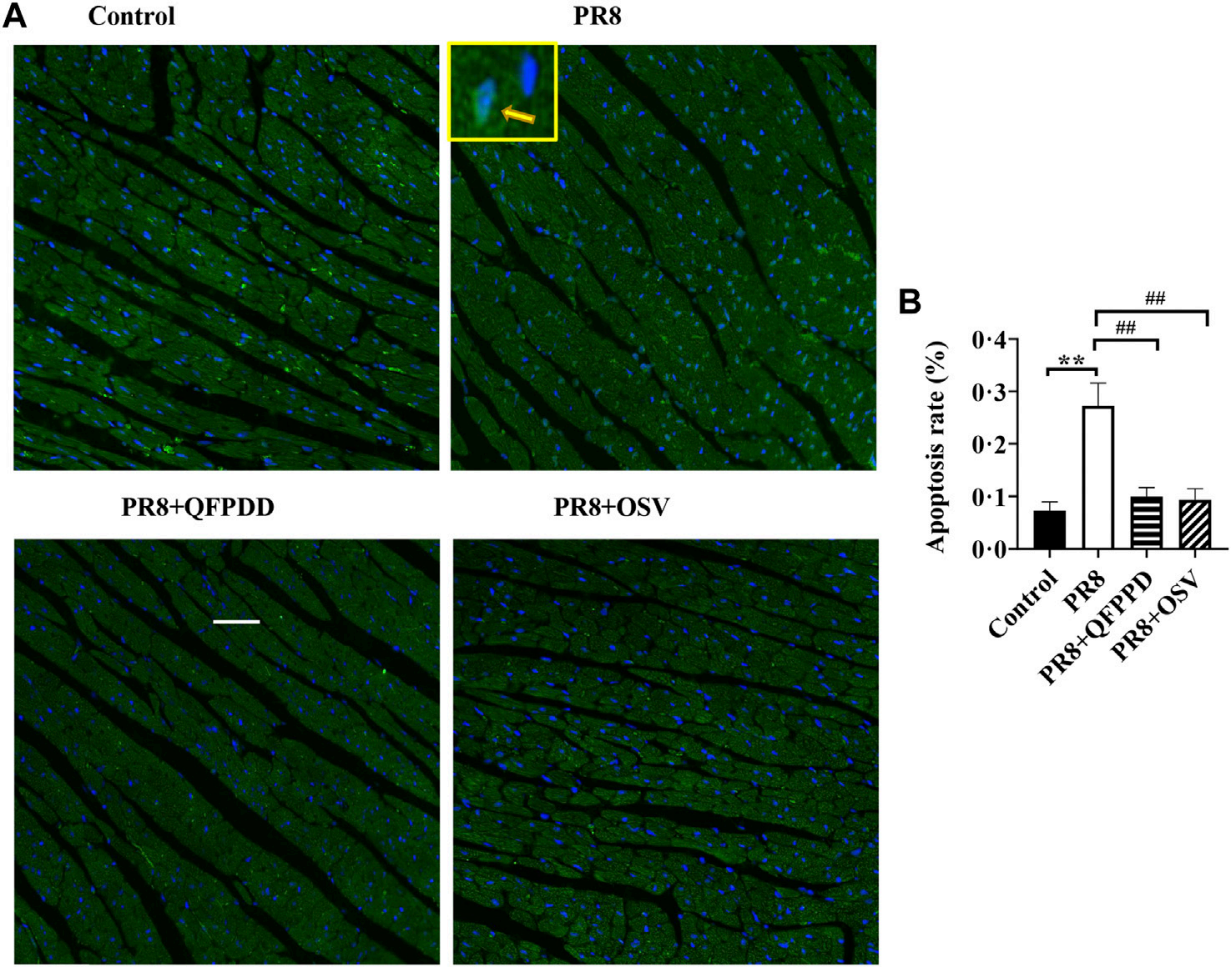
QFPDD treatment significantly inhibited the apoptosis induced by the influenza virus. **(A)** Sample immunostaining plot of TUNEL and DAPI staining in the control, PR8, PR8+QFPDD, or PR8+OSV mice. **(B)** Calculated percentage of TUNEL-positive nuclei in ventricular tissues in different groups. TUNEL-positive cells: green; nuclei: blue. Scale bar: 50 μm ^**^ *p* < 0.01 vs. the control group and ^##^ *p* < 0.01 vs*.* the PR8 group.

To determine whether QFPDD lessens the infective injury of the heart, we further examined the apoptosis of myocardial tissues using TUNEL staining. PR8 treatment significantly increased the ratio of TUNEL-positive cardiomyocytes (Figure 2; *p* < 0.01). Treatment with QFPDD or OSV significantly inhibited the increased apoptotic cell ratio in the hearts of PR8-infected mice (Figure 2; *p* < 0.01).

### 3.2 QFPDD might reduce necroptosis in cardiac tissue induced by PR8

**FIGURE 3:**
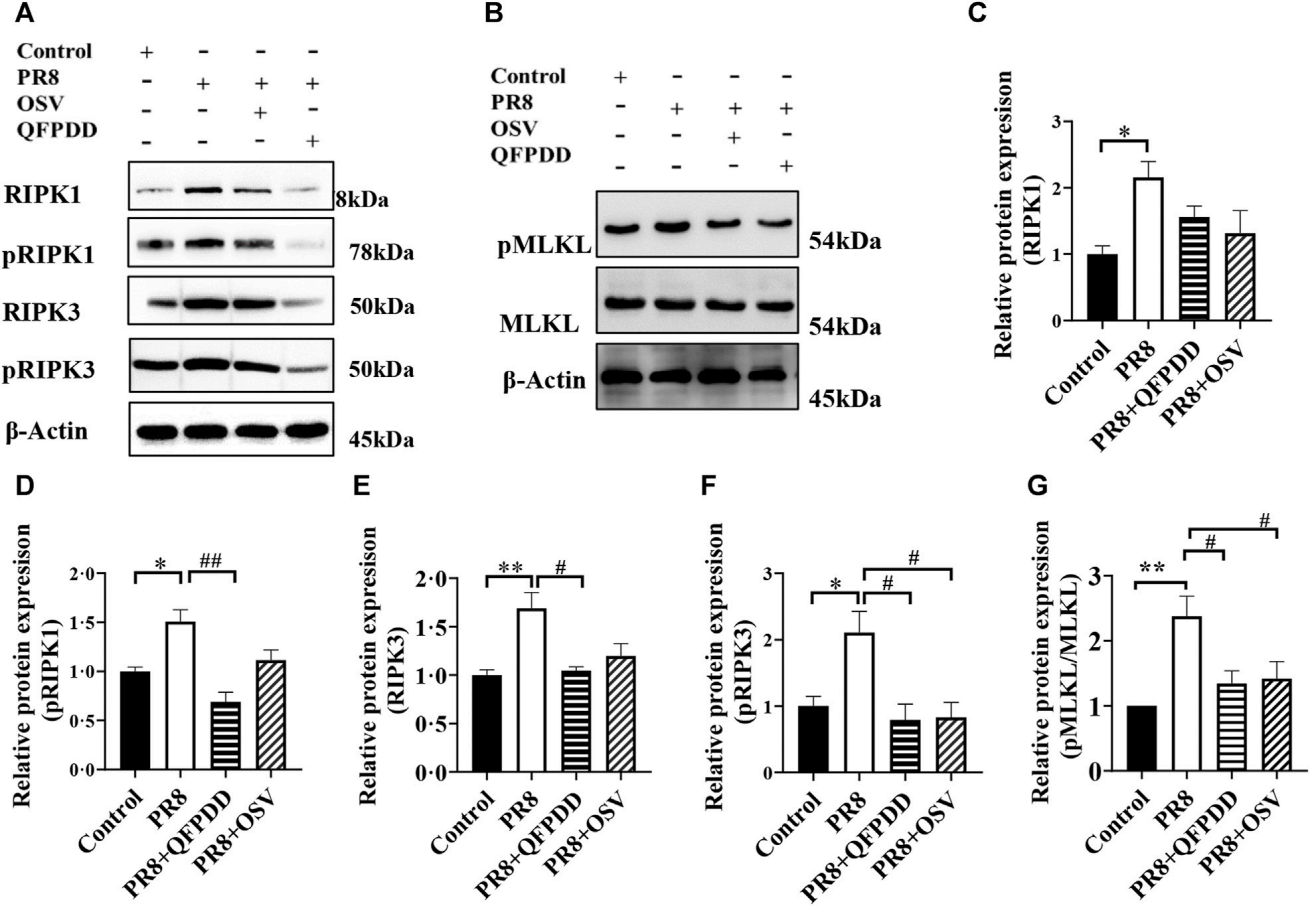
QFPDD inhibited the necroptosis of cardiomyocytes in the influenza mouse model. **(A)** Sample Western blot plot of RIPK1, pRIPK1, RIPK3, and pRIPK3 in the control, PR8, PR8+QFPDD, or PR8+OSV mouse groups. **(B)** Sample Western blot plot of pMLKL, MLKL, and β-actin in different groups. **(C–G)** Quantitative comparison of the expression level of RIPK1 pRIPK1, RIPK3, pRIPK3, and pMLKL/MLKL in different groups. *n* = 3–6; ^*^ *p* < 0.05, ^**^ *p* < 0.01, ^#^ *p* < 0.05, and ^*##*^ *p* < 0.05.

Necroptosis plays an important role in the pathogenesis of influenza. To determine whether QFPDD can inhibit necroptosis induced by the influenza virus, the expression levels and phosphorylation of necroptosis-related proteins RIPK1, RIPK3, and MLKL were detected. The RIPK1, pRIPK1, RIPK3, pRIPK3, and pMLKL/MLKL level in cardiac tissue was significantly increased by PR8 infection, and the administration of QFPDD reversed the increased pRIPK1, RIPK3, pRIPK3, and pMLKL/MLKL levels (*p* < 0.05; Figure 3). As shown in Figure 3, QFPDD can inhibit the expression of necroptotic markers of myocardial cells infected by the influenza virus, hinting that it might reduce the necroptosis of myocardial cells during influenza.

### 3.3 QFPDD treatment inhibited the expression of inflammatory factors in the myocardium of the influenza virus PR8-infected mice

**FIGURE 4:**
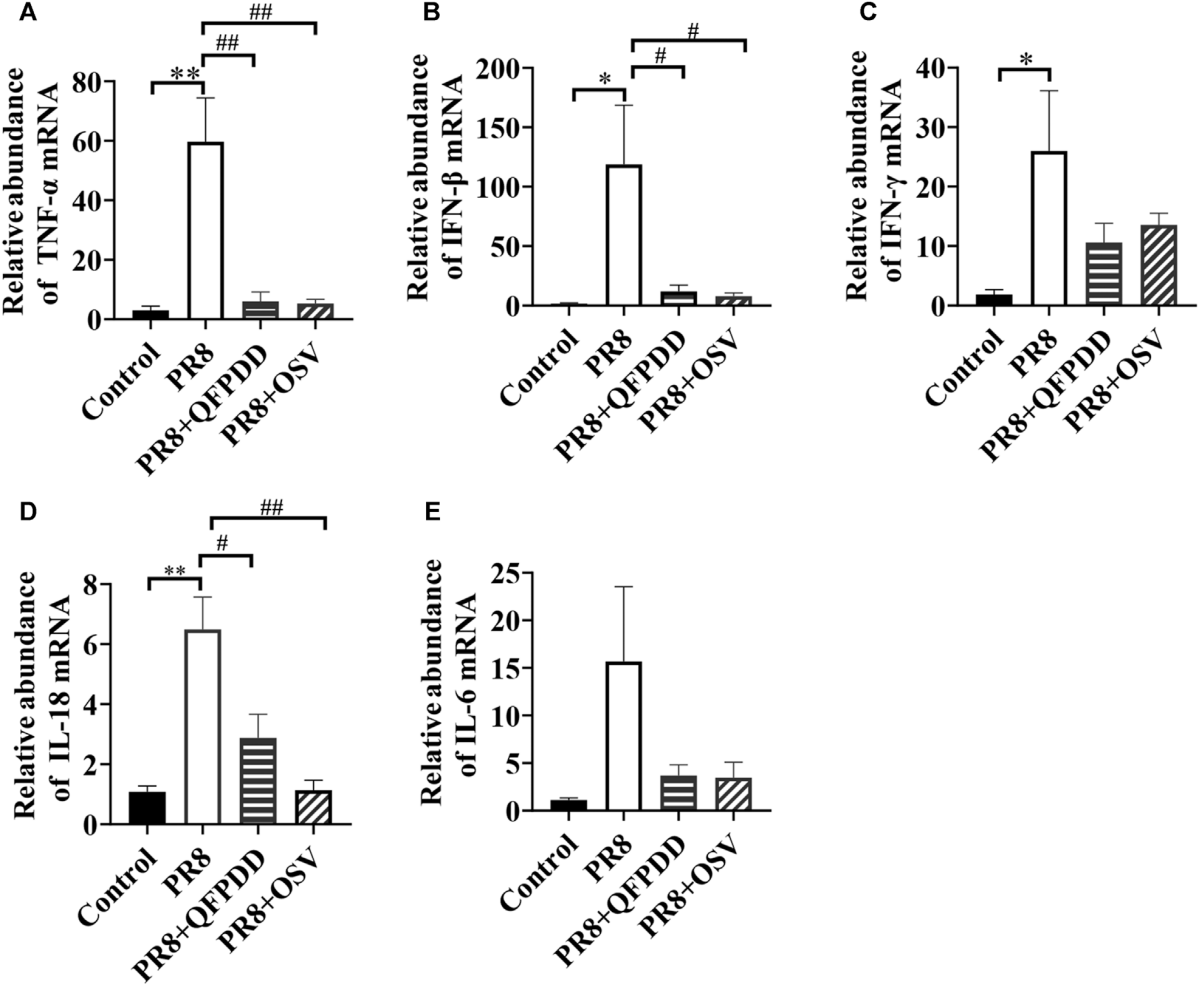
QFPDD treatment inhibited the relative abundance of inflammatory factors in the myocardium of influenza virus-infected mice. Quantitative comparison of the mRNA expression of TNF-α **(A)**, IFN-β **(B)**, IFN-γ **(C)**, IL-18 **(D)**, and IL-6 **(E)** in cardiac tissues of different groups. ^*^ *p* < 0.05 vs. the control group, ^**^ *p* < 0.01 vs*.* the control group, ^#^ *p* < 0.05 vs*.* the PR8 group, and ^##^ *p* < 0.01 vs*.* the PR8 group.

Studies have found that inflammatory factors are the main causes of tissue and organ damage during viral infection. To investigate whether QFPDD exerted its protective effects on PR8-infected cardiac tissue damage by inhibiting inflammatory responses, the expression of inflammatory factors in the myocardium was evaluated by RT-qPCR. PR8 treatment significantly increased the levels of inflammatory factors TNF-α, IFN-β, IFN-γ, and IL-18 (Figure 4; *p* < 0.05). Both QFPDD and OSV treatment decreased the expression of the inflammatory factors TNF-α, IL-18, and IFN-β in the myocardium of PR8-infected mice (Figure 4; *p* < 0.05).

### 3.4 HIF-1α is a key target for the protective effects of QFPDD in myocardial cells

**FIGURE 5:**
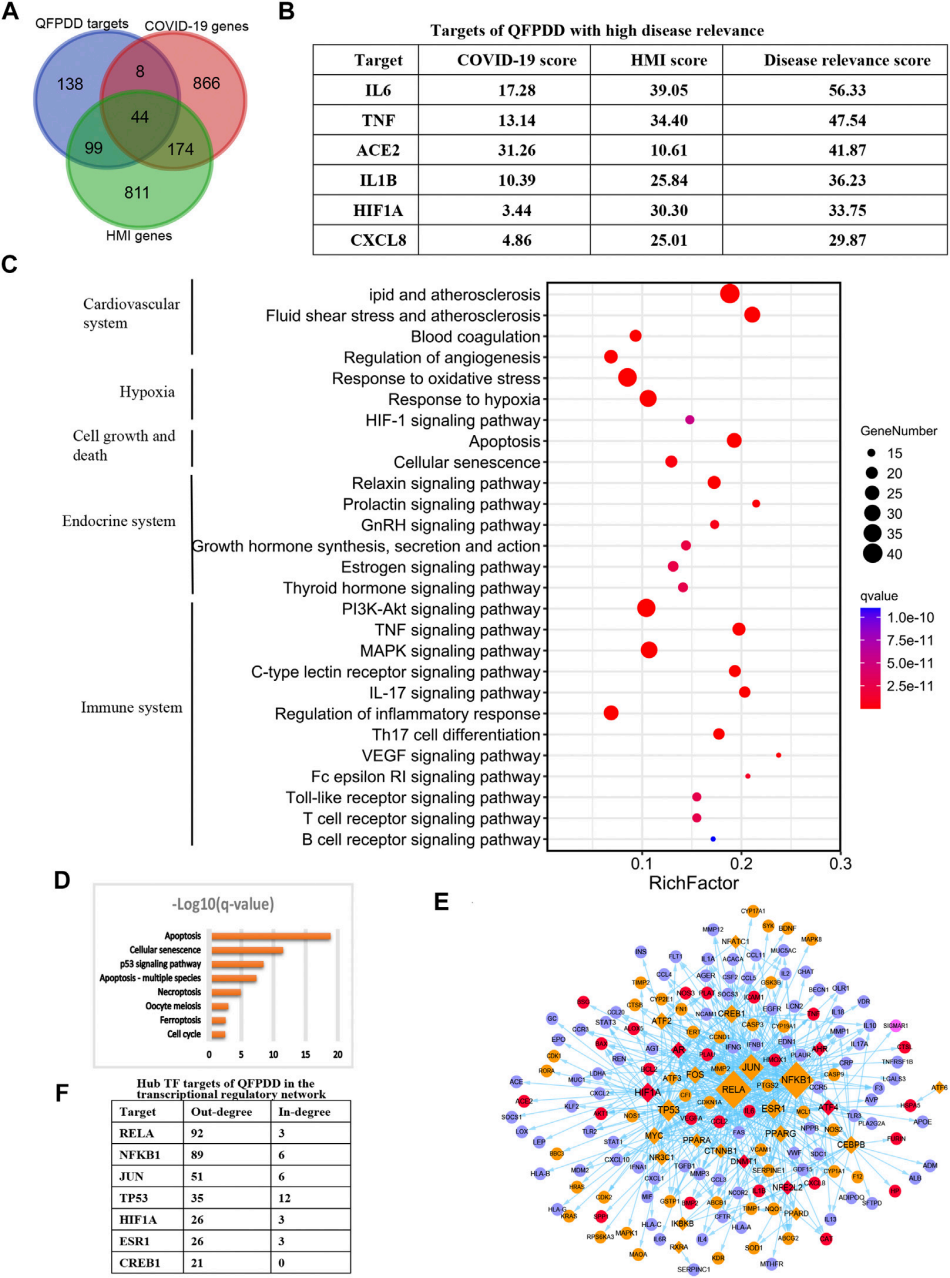
Identification of key targets of QFPDD in treating COVID-19-related cardiac damage. **(A)** Intersection between QFPDD targets, COVID-19 genes, and HMI genes. **(B)** Targets of QFPDD with high disease relevance. **(C)** Top pathways enriched with QFPDD targets associated with COVID-19 or HMI. **(D)** Pathways of cell growth and death, which are enriched with QFPDD targets. **(E)** Transcriptional regulatory network of the important targets of QFPDD to genes associated with HMI and COVID-19. Diamonds represent TFs, and circles represent targets regulated by TFs. Red nodes represent QFPDD targets, which overlap with both COVID-19 and HMI disease genes. Orange nodes represent QFPDD targets, which overlap with HMI disease genes. Pink nodes represent QFPDD targets, which overlap with COVID-19 disease genes, and blue nodes represent disease genes associated with both COVID-19 and HMI. The size of a node is proportional to its out degree. **(F)** Hub TF targets of QFPDD in the transcriptional regulatory network.

To identify the potential targets of QFPDD to reverse COVID-19-induced myocardial cell damage, the intersection between QFPDD targets, COVID-19 genes, and HMI genes was analyzed. A total of 151 targets of QFPDD were also disease genes of COVID-19 or HMI genes, accounting for 52% of all the targets (Figure 5A). These targets may play important roles in the effects of QFPDD on COVID-19-related hypoxic myocardial damage. Among the 44 targets that overlap both COVID-19 and HMI genes, 6 targets with top disease relevance scores could have high functional significance for QFPDD treatment of COVID-19-related myocardial cell damage (Figure 5B).

Functional annotation for the 151 important targets suggested that they were involved in the biological processes of the cardiovascular system, hypoxia, cell growth, and death, endocrine system, and immune system (Figure 5C; q-value < 10−10). Specifically, QFPDD regulated a series of biological processes related to cell growth and death, including apoptosis and necroptosis (Figure 5D; q-value <0.01). Then, we constructed a transcriptional regulatory network of important QFPDD targets to genes associated with HMI and COVID-19 (Figure 5E). This network included 26 transcription factors (TFs), in which the out degree of 6 TFs was larger than 20 (Figure 5F). This means that the six TFs were hub nodes of the network and had high topological significance in the network.

Integrating top functional and topological significance targets, we identified that the alpha subunit of HIF-1α was the only overlap target from both functional and topological views as the key target of QFPDD for treating COVID-19 reduced HMI.

### 3.5 Validation of the efficacy of QFPDD and its compounds to HIF-1α

**FIGURE 6:**
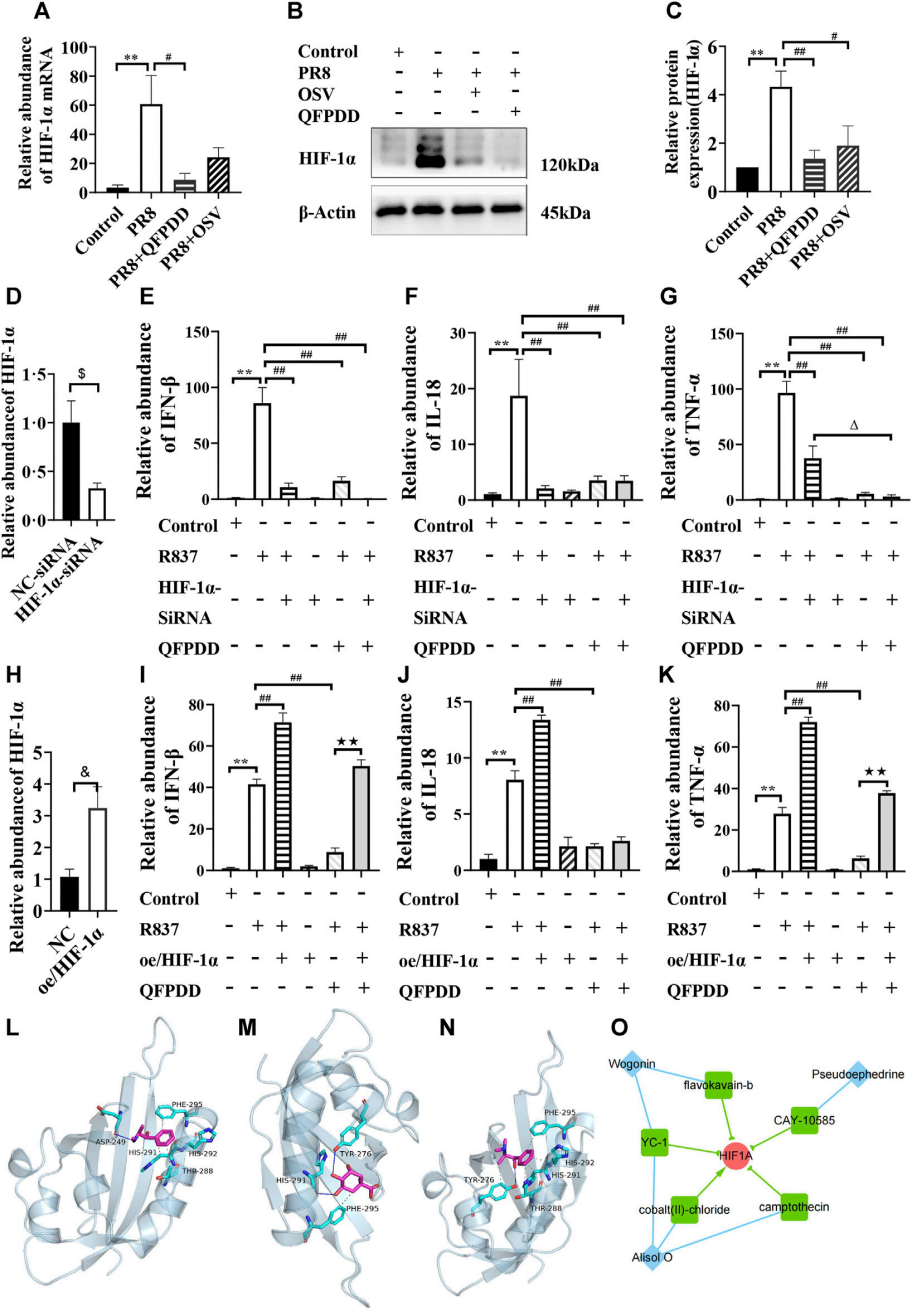
Validating the efficacy of QFPDD and its compounds on HIF-1α. **(A)** Quantitative comparison of the relative abundance of HIF-1α mRNA in the control, PR8, PR8+QFPDD, or PR8+OSV mice. **(B)** Sample Western blot plot of HIF-1α and β-actin in different groups. **(C)** Quantitative comparison of the expression level of HIF-1α in different groups. **(D)** Quantitative comparison of the relative abundance of HIF-1α mRNA in the NC–siRNA- and HIF-1α–siRNA-treated group. **(E–G)** Quantitative comparison of the relative abundance of IFN-β **(E)**, IL-18 **(F)**, and TNF-α **(G)** in the myocardial cells of different groups. **(H)** Quantitative comparison of the relative abundance of HIF-1α mRNA in the control and HIF-1α overexpression groups. **(I–K)** Quantitative comparison of the relative abundance of IFN-β **(I)**, IL-18 **(J)**, and TNF-α **(K)** in the myocardial cells of different groups. ^**^ *p* < 0.01 vs. the control group, ^#^ *p* < 0.05 vs*.* the PR8 group or R837-treated group, and ^##^ *p* < 0.01 vs. the PR8 group or R837-treated group. ^$^ *p* < 0.05 vs*.* NC–siRNA, ^&^ *p* < 0.05 vs*.* NC–plasmid, ^△^ *p* < 0.05 vs*.* the HIF-1α–siRNA and R837 co-treated group, and ^★★^ *p* < 0.01 vs*.* the QFPDD and R837 co-treated group. *n* = 4–6. **(L–N)** The interaction between QFPDD compounds pseudoephedrine **(L)**, quinic acid **(M)**, methylephedrine **(N)**, and HIF-1α protein was simulated by molecular docking (solid and dashed lines correspond to hydrogen bonds and hydrophobic bonds, respectively). **(O)** CMAP analysis suggested that three QFPDD compounds (diamond nodes) showed similar pharmacological activity with five compounds (box nodes) that target HIF-1α.

To validate the influence of HIF-1α by QFPDD, we infected mice with the influenza virus, PR8, to evoke myocardial damage. Both the mRNA abundance and the protein expression of HIF-1α were significantly increased in mice infected by PR8. QFPDD treatment significantly inhibited the increased mRNA abundance and the protein expression of HIF-1α (*p* < 0.05; Figures 6A–C).

To further demonstrate whether the HIF-1α signal is the key pathway for the protective effect of QFPDD in virus-induced heart damage, HIF-1α was knocked down or overexpressed in the cultured myocardial cells, and then, these cells were treated with R837, which is usually used to mimic the effect of single-stranded RNA-mediated inflammatory response. We found that both HIF-1α knockdown and treating the myocardial cell with QFPDD significantly reversed the R837-increased abundance of inflammatory factors (IFN-β, IL-18, and TNFα; Figures 6D–G). Co-treating the myocardial cell with QFPDD and knocking down HIF-1α did not further decrease the relative abundance of IFN-β and IL-18 (Figures 6E, F). Moreover, we found that the overexpression of HIF-1α did not change the inflammatory factors in the control group, but it significantly increased the relative abundance of IFN-β, IL-18, and TNFα in the R837-treated group and reversed the inhibition effects of QFPDD on cytokine expression (Figures 6H–K). The relative abundance of IFN-β and TNFα in the R837 and QFPDD co-treated group was significantly increased by the overexpression of HIF-1α (*p* < 0.01; Figures 6 I, K). These results indicated that QFPDD executed its protective effects partially through the HIF-1α signaling pathway.

To investigate which ingredients in QFPDD played the protective role of inhibiting HIF-1α activity, molecular docking simulation was performed to explore the binding possibility between HIF-1α and each compound of the 21 monomers from QFPDD. The three-dimensional structures of the compounds and the crystal structure of the heterodimer HIF-1α/HIF-β (pdb ID: 4h6j) were downloaded from the PubChem database and the RCSB Protein Data Bank, respectively. We extracted HIF-1α (chain A) from the dimer using PyMOL. Then, we predicted its binding site using Protein Plus. The predicted docking pocket has its center at (19.65, −9.94, and −28.9) and a diameter of 20 Å. Following that, AutoDock Vina was used to conduct the docking simulation between each compound and HIF-1α. The results suggested that five compounds of QFPDD, i.e., pseudoephedrine, quinic acid, salicylic acid, ephedrine, and methylephedrine, could bind with HIF-1α (docking scores are smaller than −4 kcal/mol). The details of binding between the compounds and HIF-1α are shown in Table 2. Three pairs of compound–target interactions are shown in Figures 6L–N.

**TABLE 2:** Binding details of QFPDD compounds with HIF-1A (pdb ID: 4h6j).

Compound	Docking score (kcal/mol)	Hydrogen bond sites of HIF-1A	Hydrophobic interaction sites of HIF-1A
Methylephedrine	−4.7	Tyr-276	Tyr-276, Thr-288, His-291, His-292, and Phe-295
Quinic acid	−4.5	Tyr-276 and His-291	Phe-295
Pseudoephedrine	−4.4	Asp-249	Thr-288, His-291, His-292, and Phe-295
Ephedrine	−4.4	Asp-249	Thr-288, His-291, His-292, and Phe-295
Salicylic acid	−4.3	Thr-288	

Next, we analyzed the gene expression profiles of MCF7 cells treated with 16 compounds from QFPDD in the CMap L1000 database. The connectivity score measures the similarity between the query profile and the reference profile in the database, which was derived from a model cell line treated with a specific chemical. A score close to 100 indicates a high degree of similarity, suggesting that the query compound may have similar pharmacological effects on cells as the matched chemical substance. By setting the threshold for the connectivity score as 70, we identified that three compounds from QFPDD (alisol O, pseudoephedrine, and wogonin) showed similarity in expression profiles with five reference chemicals in the database targeting HIF-1α. Among them, four chemicals are HIF-1α inhibitors. Therefore, the CMAP analysis suggested that the three compounds from QFPDD may have the pharmacological effects of inhibiting the activity of HIF-1α (Figure 6O).

## 4 Discussion

Increasing epidemiological and clinical evidence shows that cardiac damage is closely related to the infection of single-stranded RNA viruses, such as SARS-CoV-2 and influenza virus (Kochi et al., 2020). However, the underlying mechanism and treatment of virus-induced cardiac damage need further demonstration. In this study, we found that QFPDD treatment reduced PR8-infected myocardial cell apoptosis, significantly reducing the inflammatory response and necroptosis.

Necroptosis might be one of the mechanisms by which heart injury was exacerbated after PR8 infection, and this injury could be reversed partially by the administration of QFPDD. The activation of RIPK1 and subsequent recruitment of RIPK3 are key steps in initiating necroptosis. The activation of RIPK3 induces its self-phosphorylation and subsequently phosphorylates MLKL. Phosphorylated MLKL forms oligomers and disrupts the cellular membrane, causing cell death. The interactions and phosphorylated events among these molecules play a crucial role in regulating the necrotic pathway (Xin et al., 2023). In the current study, the levels of necroptosis-related proteins were increased in PR8-infected mouse cardiomyocytes, while QFPDD treatment significantly inhibited the necroptosis of cardiomyocytes in PR8-infected mice, which further confirmed the contribution of necroptosis to influenza virus-induced cardiac injury.

Influenza virus pathogenesis has been strongly associated with a robust production of cytokines, referred to as the “cytokine storm.” Viral infection triggers the innate immune response of the host, which constitutes the first line of defense against invading pathogens (Shi et al., 2020; Shi et al., 2021; Ye et al., 2023). Traditional Chinese medicine may protect different organs from viral infection by regulating the immune responses (Yang et al., 2020; Shi et al., 2021). Our previous study has demonstrated that QFPDD reduced the lung indexes and downregulated the expression of MCP-1, TNF-α, IL-6, and IL-1β in lungs or serum samples. QFPDD may protect the host from an overwhelming inflammatory response by decreasing the high M1/M2 ratio of macrophages (Ye et al., 2023). Inflammatory factors have also been associated with cardiovascular diseases and proposed as cardiovascular risk biomarkers (Bullón et al., 2017). Our result indicated that QFPDD may exert its protective effects on myocardial cells through inhibiting the virus-mediated expression of inflammatory factors, such as IFN-β, TNF-α, and IL-18. The increased inflammation in cardiac tissue might be induced by increased inflammation factors in blood and through the direct effect of the virus in cardiac (An et al., 2021; Bhat et al., 2021; Ye et al., 2023). We found that influenza viruses can translocate to cardiac tissue, which is consistent with the results of other studies that human influenza viruses can infect cardiomyocytes in the mouse heart (Bhat et al., 2021). The virus in cardiac tissue might partially modulate the local inflammatory response directly (An et al., 2021).

HIF-1 is a heterodimer composed of α and β subunits and a key transcription factor in the cellular response to hypoxia (Xin et al., 2023). In our study, HIF-1α was identified as a possible key target of QFPDD in the treatment of virus-induced myocardial injury. QFPDD might protect the cardiac tissue by downregulating the expression of inflammatory cytokines at least partially by inhibiting the upregulation of HIF-1α expression. Our results indicated that the HIF-1α level was significantly increased by PR8 infection. Both HIF-1α knockdown and treating the myocardial cell with QFPDD significantly reversed the increased inflammatory factors (IFN-β, IL-18, and TNF-α) mediated by viral infection. The overexpression of HIF-1α reversed the inhibition effects of QFPDD on cytokine expression. However, we could not exclude the possibility that HIF-1α also plays a role in viral infection. A few viral particles were also found in the cardiac tissue in the PR8-infected group but not in the QFPDD-treated group. HIF-1α has been demonstrated to promote the replication of viruses in monocytes (Tian et al., 2021); thus, it is possible that inhibiting HIF-1α might also blunt the viral replication. The molecular docking and CMAP analysis indicated that seven compounds from QFPDD (pseudoephedrine, quinic acid, salicylic acid, ephedrine, methylephedrine, alisol O, and wogonin) may play the role of targeting HIF-1α. Currently, there is a lack of crystal structures documenting the binding of a ligand to HIF-1α. As a result, we utilized Protein Plus to predict potential binding pockets. The molecular docking analysis revealed that five compounds from the QFPDD dataset exhibited binding scores lower than −4 kcal/mol with HIF-1α. Additionally, a sufficient number of hydrogen bonds and hydrophobic interactions were observed between the ligand and receptor. These findings indicated stable interactions between HIF-1α and the compounds. Thus, QFPDD might inhibit virus-induced cardiac damage through the HIF-1α pathway.

**FIGURE 7:**
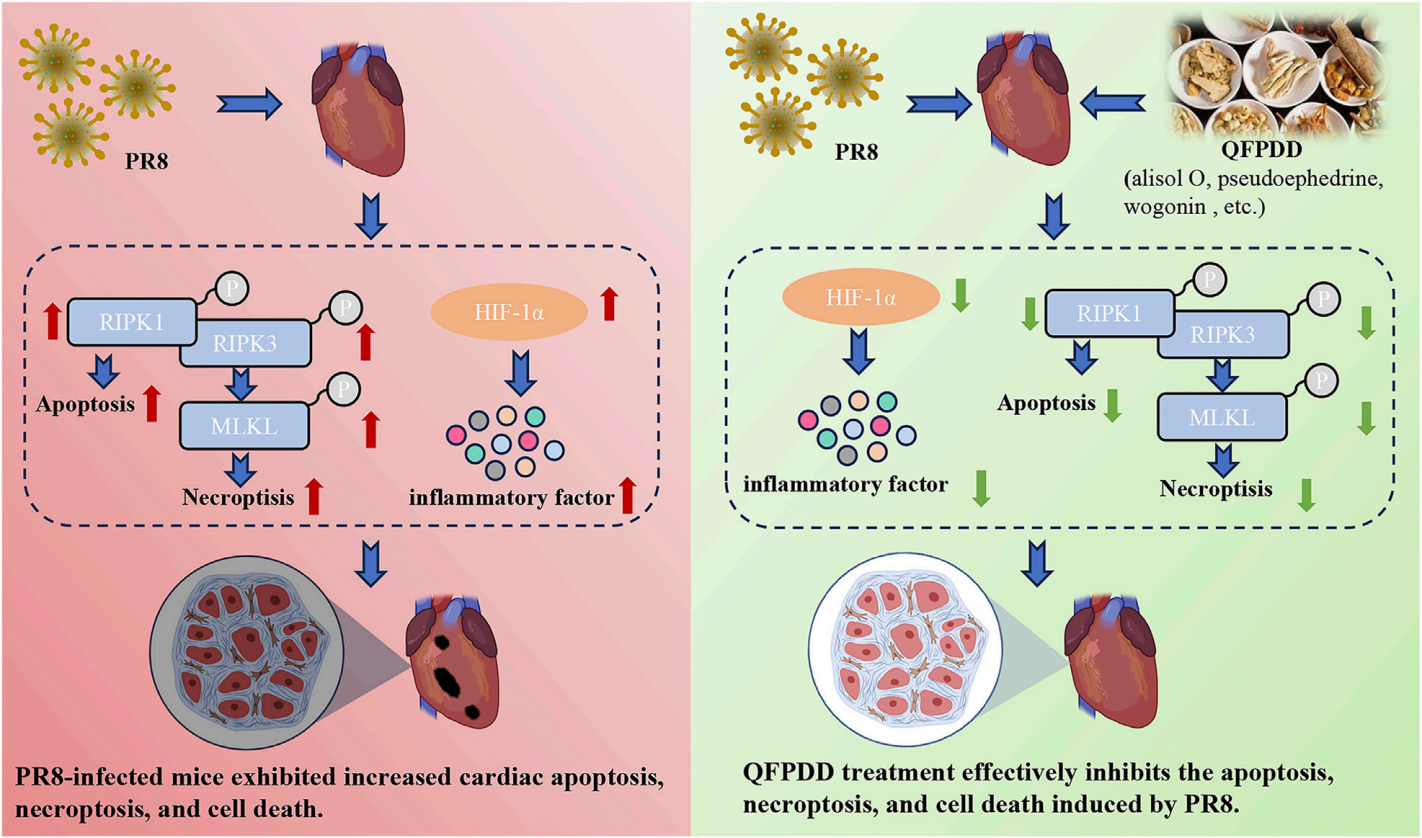
Effect and mechanism of QFPDD in the management of PR8-infected cardiac tissue damage. The current results showed that QFPDD could prevent the cardiac injury caused by influenza virus infection by reducing the degree of cell necroptosis, reducing inflammatory response, and HIF-1α is a key target for the beneficial effects of QFPDD in the treatment of PR8-infected induced cardiac damage.

In conclusion, the current results showed that QFPDD could prevent cardiac injury caused by influenza virus infection by reducing the degree of cell necroptosis and reducing the inflammatory response, and HIF-1α is a key target for the beneficial effects of QFPDD in PR8-infected cardiac damage (Figure 7). These findings may provide new ideas for the treatment of influenza or COVID-19-related myocardial damage.

## Data Availability

The original contributions presented in the study are included in the article/Supplementary Material; further inquiries can be directed to the corresponding authors.
